# Alterations in gene expression in *Caenorhabditis elegans* associated with organophosphate pesticide intoxication and recovery

**DOI:** 10.1186/1471-2164-14-291

**Published:** 2013-04-30

**Authors:** John A Lewis, Elizabeth A Gehman, Christine E Baer, David A Jackson

**Affiliations:** 1 US Army Center for Environmental Health Research, Fort Detrick, MD, USA; 2 Current Address: The MITRE Corporation, McLean, VA, USA; 3 Excet Inc., Fort Detrick, MD, USA

**Keywords:** Caenorhabditis elegans, Organophosphate pesticide intoxication, Gene expression, Dichlorvos, Acetylcholinesterase inhibition, Mitochondrial disruption, Dichlorvos-induced developmental delay

## Abstract

**Background:**

The principal toxicity of acute organophosphate (OP) pesticide poisoning is the disruption of neurotransmission through inhibition of acetylcholinesterase (AChE). However, other mechanisms leading to persistent effects and neurodegeneration remain controversial and difficult to detect. Because *Caenorhabditis elegans* is relatively resistant to OP lethality—particularly through the inhibition of AChE—studies in this nematode provide an opportunity to observe alterations in global gene expression following OP exposure that cannot be readily observed in less resistant organisms.

**Results:**

We exposed cultures of worms in axenic, defined medium to dichlorvos under three exposure protocols. In the first, worms were exposed continuously throughout the experiment. In the second and third, the worms were exposed for either 2 or 8 h, the dichlorvos was washed out of the culture, and the worms were allowed to recover. We then analyzed gene expression using whole genome microarrays from RNA obtained from worms sampled at multiple time points throughout the exposure. The worms showed a time-dependent increase in the expression of genes involved in stress responses. Early in the exposure, the predominant effect was on metabolic processes, while at later times, an immune-like response and cellular repair mechanisms dominated the expression pattern. Following removal of dichlorvos, the gene expression in the worms appeared to relatively rapidly return to steady-state levels.

**Conclusion:**

The changes in gene expression observed in the worms following exposure to dichlorvos point towards two potential mechanisms of toxicity: inhibition of AChE and mitochondrial disruption.

## Background

Because organophosphate (OP) pesticides are widely used for the control of agricultural pests and of arthropod disease vectors commercially, residentially, and institutionally, large numbers of people throughout the world are routinely exposed to OPs, many at toxic levels. It has been estimated that there may be as many as three million poisonings and 200,000 deaths per year from OP exposure worldwide [1].

All OPs exert their primary effects as acetylcholinesterase (AChE) inhibitors. Both short term and long-term adverse effects of AChE inhibitor exposure have been described, ranging from acutely life-threatening conditions to subtle, long term behavioral deficits. However, the consequences of low level exposure and the nature and mechanism of persistent effects are poorly understood and, in many cases, controversial (see below).

Without intervention, high-dose, acute exposures to OPs and other AChE inhibitors result in death from respiratory failure; less severe exposures may cause excessive salivation, lacrimation, and urination, as well as diaphoresis, gastrointestinal motility, and emesis followed by paralysis [2]. People and animals that recover from acute exposures to many of these compounds may present with a delayed syndrome; organophosphate induced delayed polyneuropathy (OPIDP), characterized by the appearance of numbness, weakness, and parathesia in the limbs, and degeneration of peripheral nerve and central nervous system myelin sheaths 7–21 days after exposure [3-5]. A so-called intermediate syndrome, which presents 24–96 h after exposure, has been identified and is characterized by weakness of the neck, proximal limb, and respiratory musculature. This syndrome is believed to result from acetylcholine receptor desensitization [4,5]. The occurrence of persistent neurological and neuropsychiatric effects after low level exposure and of developmental neuro-behavioral effects has also been described [6-8]. These effects remain controversial, however, [4,6,9] because of the difficulty in obtaining persuasive evidence of low-level and transient exposures to these compounds [10].

In an effort to resolve some of the issues surrounding acute, transient, and low-level exposures to OPs, we have undertaken studies tracking both development of and recovery from OP intoxication at the global gene expression level using the genomic model organism *Caenorhabditis elegans* and Affymetrix whole genome *C. elegans* GeneChip microarrays. *C. elegans* shows substantial similarity to mammals in the relevant biochemistry and genomics. The acute toxicity of the commercial OPs, including dichlorvos, results from inhibition of acetylcholinesterase in vertebrates [11], and this mechanism appears to be the same in worms as well [12]. In contrast to mammals, however, the nematode possesses four AChE genes rather than a single alternatively spliced gene [13]. Mutations in at least 18 *C. elegans* genes have been shown to confer resistance to the OP-like carbamate pesticide aldicarb, and all these resistance genes are known—or plausibly believed—to be involved in acetylcholine metabolism, secretion, or recycling [14]. The *C. elegans* genome also contains a close homolog of the vertebrate secondary OP target, neuropathy target esterase (NTE) [15]. Impairment of NTE function is thought to underlie OPIDP [3,5,16].

In previous work, we found that *C. elegans* is relatively resistant to OP lethality [17] but incurred developmental abnormalities after 24 h in high concentrations of the AChE inhibitors aldicarb (a carbamate), fenamiphos, and dichlorvos (OPs; unpublished observations). The abnormalities included malformed cuticles, severe anatomical disorganization, and protruding vulvae, suggesting that the OPs also target molecules other than AChE. A large number of non-AChE and non-NTE targets have been proposed in humans and other vertebrates, including nicotinic, muscarinic, and cannabinoid receptors, kinases, and carboxylesterases in addition to AChE and NTE [4,5,11]. The potent neurotoxicity of the OPs, however, has made it difficult to study “off-target” effects.

We chose to perform the present studies with dichlorvos in order to maintain comparability with previous work and also because dichlorvos is known to reversibly inactivate AChE [18]. We reasoned that it might be possible to wash dichlorvos out of the worm culture so that the worms’ recovery from acute intoxication could be followed. While it is unlikely that *C. elegans* experiences a syndrome like OPIDP because of the brevity of its life-span and the shortness of its neurons [16], OPIDP has been described in humans following dichlorvos exposure [19]. This raises the possibility that the consequences of NTE inhibition by dichlorvos might nevertheless be observable in *C. elegans* at the gene expression level. As noted above, we have observed developmental abnormalities in worms exposed to dichlorvos suggesting that there could be undiscovered targets for OPs in *C. elegans.*

**Figure 1:**
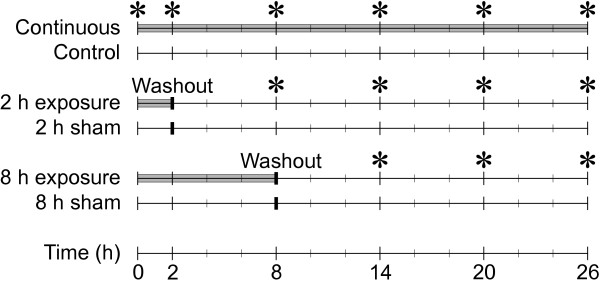
**Experimental design for OP acute time course experiment.** Worms were cultured in medium containing 15.0 μM, 0.6 μM, or no dichlorvos. Three exposure regimes were used. In the first, the worms remained in medium until the time of harvest. In the two others, the toxicant was washed out at 2 or 8 h after initiation of the experiment. Sham exposures are untreated controls that have been subjected to the washout procedure. Asterisks indicate when samples were taken.

Because *C. elegans* is relatively resistant to OP lethality, yet shows substantial similarity to mammals in the principal biochemical systems targeted by OPs, we conjectured that it might be possible to highlight non-cholinergic effects from OP exposures in the worm that are difficult to discern using animal models less resistant to OPs. We either exposed synchronized L3/L4 stage *C. elegans* cultures to dichlorvos or washed the dichlorvos out of the culture to allow the worms to recover (Figure 1). We harvested worms at several time points and performed an analysis of global gene expression. In these studies, we have elucidated perturbations in key pathways related to energy metabolism, innate immunity, and cellular damage resulting from OP exposure.

**Table 1:** **Behavior of *C. elegans *** **following dichlorvos exposure**

**Dichlorvos (μM)**	**Continuous exposure**		**4 h wash-out**	
	**Reduced movement**	**24 h**	**3 h post washout**	**20 h post washout**
15.0	10 min	immobile	reduced motility	motility returned to control levels
3.0	40 min	immobile	reduced motility	motility returned to control levels
0.6	4 h	sluggish	motility returned to control levels	motility returned to control levels
0.12	NOE	NOE	NOE	NOE

Worms were exposed to various concentrations of dichlorvos either continuously for 24 h or for 4 h followed by removal of the dichlorvos and recovery in fresh medium for 3 or 20 h. Worms were examined microscopically, and the amount of movement compared with controls was recorded.

## Results and discussion

Organophosphate (OP) pesticides are commonly used and may account for as many as three million poisonings and 200,000 deaths per year worldwide [1]. The principal mechanism of acute toxicity, inhibition of acetylcholinesterase (AChE), has been well studied, yet little is known about the cause of “off-target” effects, including persistent and delayed neurological effects. To investigate off-target effects, we chose the nematode, *C. elegans*, because of its resistance to lethality from AChE inhibition. Range-finding experiments were performed to determine suitable exposure concentrations, and worms were exposed to two concentrations dichlorvos. In a subset of the cultures, the toxicant was removed to examine recovery specific mechanisms. Gene expression changes were monitored throughout the course of the experiment using whole genome microarrays. A striking consequence of the exposure was delayed development (see below), which complicated analysis because of the interplay between developmentally-regulated events and toxicant-induced expression changes. In general, the worms showed time-dependent responses to the toxicant, which progressively intensified. The major effects of the exposure included metabolic disruption, and innate immunity-like and cellular repair responses.

### Effects of dichlorvos on worm motility

**Figure 2:**
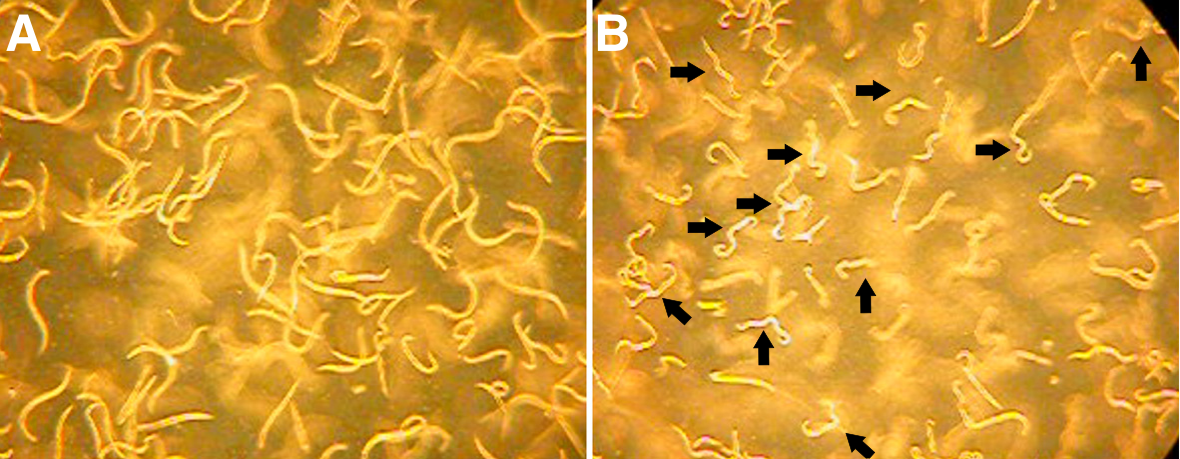
**Effect of 1 h exposure to 15 μM dichlorvos on *C. elegans *body conformation. A**) Unexposed stage L4 worms. **B**) L4 worms exposed to 15 μM dichlorvos for 1 h. Note the sharp bends in the worms (arrows). Screen captures from digital video.

We performed range-finding experiments by observing the effects of varying concentrations of dichlorvos on motility since impairment of worm movement is a readily observable consequence of OP exposure in worms (Table 1) [12,14,20]. For the definitive experiments, we chose concentrations of dichlorvos that produced observable effects on motility yet allowed the worms to recover following washout. Commencing at the early L4 stage (46 h), worms were exposed for 24 h to a series of 5-fold dilutions of dichlorvos beginning with 15 μM, a concentration with effects on gene expression at 8 h of exposure, as we have previously described [17]. At the end of the exposure, the worms cultured with 15.0 and 3.0 μM dichlorvos were immobile. Immobility was associated with the worms assuming a static form with multiple sharp bends (Figure 2), consistent with hypercontracted paralysis resulting from excess acetylcholine at the neuromuscular junction [21] and resembling the sharp bends observed by Mahoney *et al*. in worms exposed to aldicarb, a carbamate inhibitor of acetyl cholinesterase [22].

Although the rate at which worms writhed when exposed to 0.6 μM dichlorvos was visibly slowed at 4 h post-exposure, few, if any, worms became sharply bent or completely immobile even after 24 h of exposure. There was no obvious difference between control cultures and cultures exposed to 0.12 μM even after 24 h of exposure. These observations are consistent with those of Cole and coworkers [12], who developed a software package to quantify worm mobility which demonstrated that a 4 h exposure to 0.7 μM dichlorvos reduced worm motion by 50%.

To verify the reversibility of the effects of dichlorvos exposure, time-course experiments were performed in which early L4 (46 h) worms were exposed to varying concentrations of dichlorvos for 4 h, and then the culture medium was changed to remove the dichlorvos. By visual inspection, worms exposed to 3.0 μM or 15.0 μM dichlorvos showed an incomplete recovery over 3 h. In contrast, worms exposed to 0.6 μM dichlorvos appeared to be fully recovered by 3 h. By 20 h, we could not readily distinguish the behavior of the worms in any of the exposures from control. There was no discernible effect of 0.12 μM dichlorvos on the worms under any condition.

Although the worms resumed normal behavior based on visual observations, AChE activity may still have been inhibited. In studies of the recovery of adult *C. elegans* from a 24 h exposure to the AChE inhibitory pesticides carbofuran and fenamiphos, the recovery of AChE activity was incomplete even when behavioral evidence indicated complete recovery [20].

### Gene expression analysis of dichlorvos exposure

**Figure 3:**
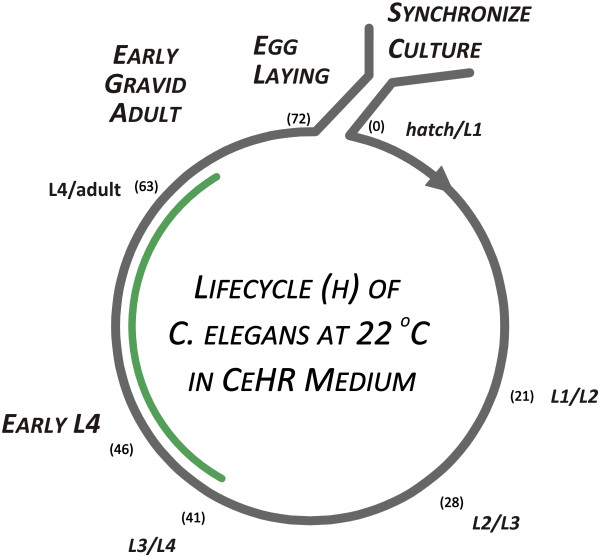
**Significant developmental markers and times for synchronized *C. elegans *cultures.** Cuticular molts between the 4 larval stages (L1-L4) and the adult worm stage are shown in *italics*. Hours post-release from the synchronization procedure are shown in parentheses. Some significant developmental events and periods are also indicated. The green arc indicates the period of the experiment.

For the definitive study, worms were exposed to two concentrations of dichlorvos; 0.6 μM—the LOEL (lowest observed effect level) determined in the motility assays—and 15 μM—a concentration previously shown to elicit changes in gene expression [17]. The exposures began at the L3/L4 stage cuticular molt (~41 h post-synchronization) and continued for 26 h, which permitted a full 18 h recovery after an 8 h exposure but did not extend into the egg-laying period (~ 72 h; Figure 3). Three exposure regimes were utilized (Figure 1). In one, there were no changes in the medium prior to harvesting the samples. In the other two, the medium was washed out and replaced with fresh medium after 2 or 8 h to remove the dichlorvos, allowing the worms to recover; control flasks were also subjected to the washout procedure (shams). Samples were harvested at the zero time point, 2 h, and then every 6 h until the conclusion of the experiment at 26 h. The entire exposure protocol was completed four times on separate days to be used as biological replicates for the microarray analysis.

Levels of expression were determined for approximately 22,500 genes represented on the whole genome microarray for each of the 148 samples; two samples failed to provide data that passed quality control standards. A total of 14,398 probe sets were detected above background levels in all the replicates of at least one condition. We considered only these probe sets in subsequent analyses. In the initial evaluation of the data, it was clear that the 2 h washout regime and the low dose exposures produced few detectable effects; therefore, we focused our investigation on the other conditions.

### Dichlorvos-dependent delayed development

**Figure 4:**
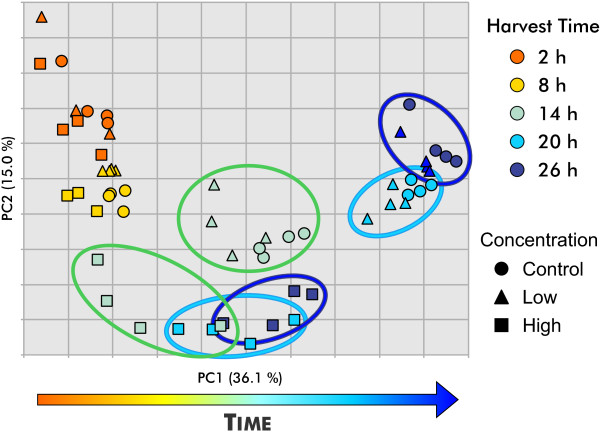
**Principal components analysis shows developmental delays in worms continuously exposed to dichlorvos.** Analysis of 14,398 probe sets present above signal to noise threshold reveals compression along PC1. Displacement along PC1 is well correlated with time. Concentrations are indicated by shapes and harvest times are indicated by color.

Previous work [17] and preliminary data exploration clearly indicated that exposure to dichlorvos causes a delay of development in the L4 larvae as judged by gene expression. A principal components analysis (PCA) of the samples from the continuous exposure protocol (Figure 4) shows that the largest effect in this experiment is associated with the time of harvest, presumably resulting from changes in gene expression throughout normal development. The spread of the clusters of worms harvested at different times in the high concentration exposure regime is compressed along the first principal component (PC1) toward the early harvest times for the unexposed samples, illustrating the dichlorvos-dependent developmental delay that complicates the analysis of these data.

**Table 2:** Predicted age

**Harvest**	**Probe sets**	**Predicted age (h)**			
		**Continuous unexposed**	**Washout unexposed**	**Continuous exposed**	**Washout exposed**
8	443	7.9 ± 0.38		4.5 ± 1.2*	
14	635	14 ± 0.27	13 ± 0.94	9.6 ± 2.4*	9.5 ± 1.5*
20	1113	19.6 ± 0.37	19 ± 0.95	14 ± 1.9*	14.7 ± 1.4*
26	361	25.8 ± 0.13	25 ± 0.23*	18.7 ± 1.3*	20.3 ± 2.7*

**Figure 5:**
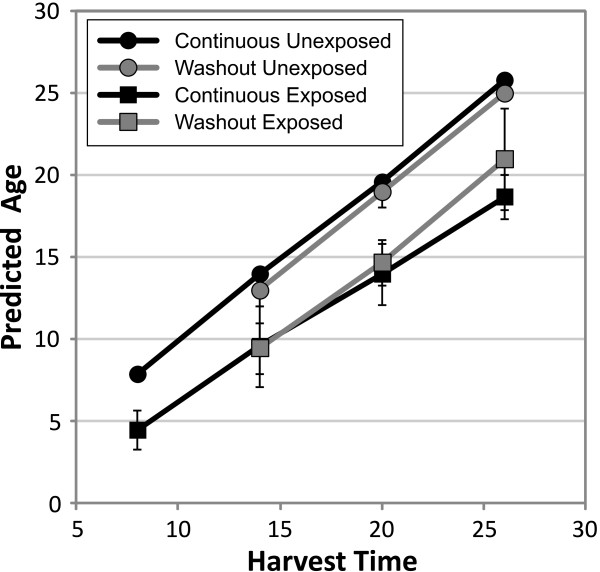
**Dichlorvos exposure shifts the timing of development.** The Predicted Age of the culture was calculated from developmental gene expression levels as described in the text and plotted against the harvest time. Dichlorvos exposure primarily affects development in the first 8 h following the L3/L4 molt and continuous exposure causes only a modest additional effect on the rate of development.

The Predicted Age of worms cultured under the continuous or the 8 h washout conditions described in the text was calculated as described in Methods; the age is calculated from the initiation of the exposure. The number of probe sets used for predicting each age is shown, and Predicted Ages are reported ± SEM. Statistically significant differences from control are indicated by asterisks. The data presented in this table are also plotted in Figure 5.

In an attempt to better understand the effects of exposure on development and to predict the amount of developmental delay, we developed a linear regression-based method for computing a Predicted Age (PA) for comparison purposes based on the expression level of developmentally-regulated genes and the time of harvest (see Methods). For the PA, the beginning of the exposure is set as the zero time.

As expected, the unexposed samples in both the continuous regime and washout regime have PAs that are similar to their harvest times (Table 2 and Figure 5). The washout procedure itself somewhat delayed development, but the effect is only significant in the 26 h-harvest condition. However, the exposed samples show significant differences from the harvest time for all times 8–26 h post-exposure. In the 20 h- and 26 h-harvest samples, there is approximately a 5–7 h delay in development in the exposed worms. Surprisingly, the worms in the continuous exposure and 8 h washout protocols showed similar developmental delays (Figure 5), suggesting that some key developmental process(es) in early L4 larvae are susceptible to interference by dichlorvos or that the cause of the delay persists at least for a time after the dichlorvos is removed.

While the profiles of the majority of the developmentally regulated genes are consistent with a well-regulated but delayed developmental process, we identified 13 developmentally-regulated genes (15 probe sets; Additional file 1) that do not appear to be affected by the developmental delay. These genes were identified by filtering the list of 6,132 developmentally regulated genes (see Methods) using the following inclusion criteria applied to data from the continuous exposure protocol: 1) a ≤ 1.2-fold difference in expression level between exposed and control conditions at each time point; 2) a ≥ 1.8-fold difference between at least one pair of consecutive harvest times in the 8–26 h time range. The only significantly enriched biological classification identified for this set was the presence of two genes that encode proteins involved in amino acid transport and metabolism (DAVID [23]; *p* = 0.045). Based on the limited annotation available for these genes, we cannot provide a clear biological interpretation of the role this small set of genes plays in development. It is also possible that the genes are regulated by both dichlorvos exposure and development in such a way that some interaction between the two makes it appear that the expression profile of these genes is not delayed.

Dichlorvos exposure significantly delayed worm development—by more than 7 h at the 26 h-harvest point as judged by the PA value. Overall, the coordinated progression of development does not appear to be disrupted, but we are unable to rule out desynchronization of a small number of developmental processes.

### Biological responses to dichlorvos exposure

**Figure 6:**
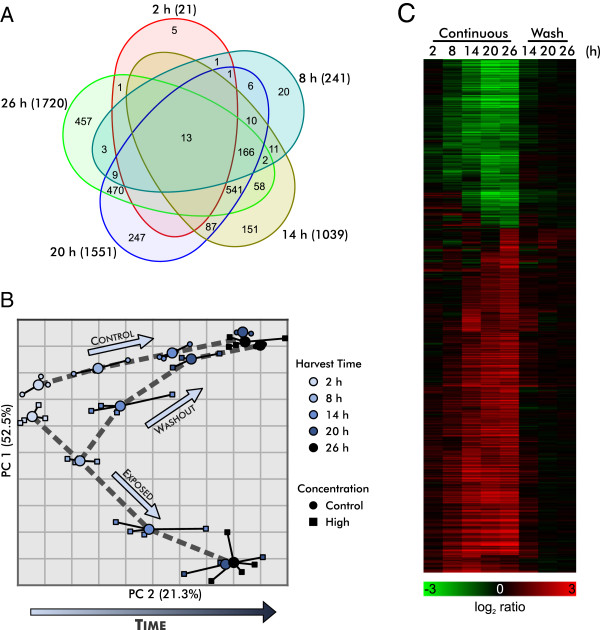
**Magnitude of effect from exposure to 15 μM dichlorvos increases with time. A**) The Venn diagram of the probe sets grouped by harvest time illustrates the overlap in gene expression and increasing number of differentially expressed genes at later harvest times. **B**) A principal components analysis of the 2259 probe sets highlights the large exposure and developmental effects. Washout samples exposed for only 8 h return to baseline. Concentrations are indicated by shapes and harvest times are indicated by color. Large circles indicate the centroid for each condition. PC2 is well correlated with time of development. **C**) A heatmap of 2259 differentially expressed probe sets shows changes (log_2_ ratio) in gene expression in worms exposed to 15 μM dichlorvos versus unexposed worms at the same predicted ages. Continuous and 8-h washout (Wash) conditions are shown.

In order to understand the mechanism of dichlorvos toxicity, we attempted to identify gene expression changes that were associated with dichlorvos exposure but were not the consequence of developmental delay. We initially limited our analysis to the continuous exposure regime and compared data from high concentration samples to control samples. Differentially expressed probe sets were selected using a *p* < 10^−4^ (FDR < 0.001) and 1.8-fold change-filter based on an analysis of covariance (ANCOVA) that included PA (Table 2) and exposure as factors. A total of 2,259 probe sets was identified across the five harvest times, with increasing numbers at later time points (Figure 6A). The expression levels of these genes increasingly diverged from control over time, but as expected, following washout, their expression rapidly returned to control levels (Figure 6B and C).

To determine the biological processes affected by exposure, we compared this list of differentially expressed genes to lists of genes or probe sets from several data sources including Gene Ontology Terms and Microarray Expression Clusters from WormBase, and transcription factor binding sites from the literature [24] (See Additional files 2, 3, 4). The major processes identified in these analyses are metabolic regulation, innate immunity, stress response, and muscular and neurological regeneration.

#### DAF-16**-**mediated stress responses

**Figure 7:**
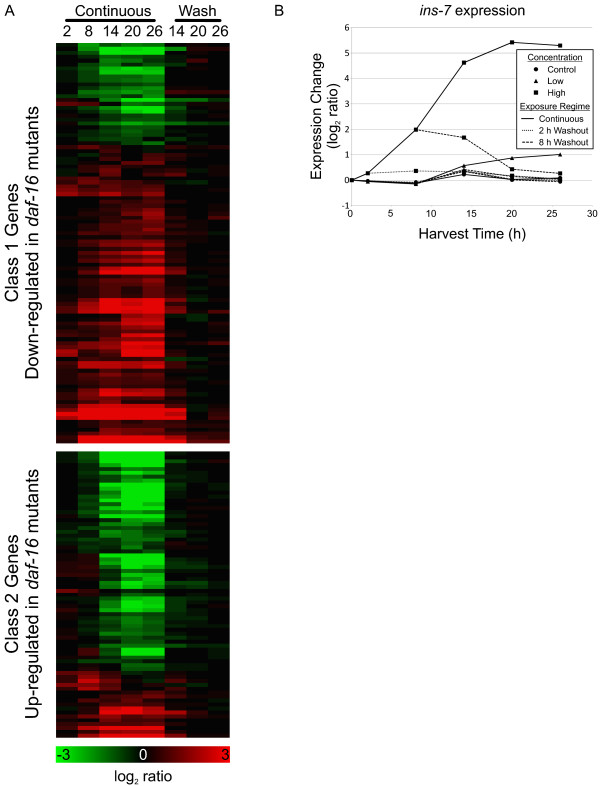
**DAF-16 target genes are differentially regulated despite over-expression of the gene encoding the DAF-2 agonist INS-7. A**) Heat map of genes downstream of DAF-16 that are known to be up-regulated or down-regulated in DAF-16 mutants [25]. Changes in gene expression (log_2_ ratio) are shown for continuous and 8-h washout (Wash) conditions. **B**) Graph of *ins-7* expression.

A large number of the genes differentially expressed in reaction to dichlorvos exposure are transcriptionally regulated by DAF-16, a forkhead transcription factor implicated in the control of metabolism, ageing, innate immunity, and stress responses in *C. elegans*. The microarray cluster enrichment analysis in particular revealed a large over-representation of genes known to be controlled by DAF-16 (Additional file 2) [25]. Murphy *et al.*[25] identified genes downstream of *daf-16* in microarray experiments using adult *daf-2*; *daf-16* hypomorphic mutant worms or worms in which DAF-2 and DAF-16 expression was knocked down with RNAi. In our work, we observed changes in expression of these downstream genes that are largely consistent with increasing DAF-16 activity over the course of the exposure (Figure 7A).

The means by which DAF-16 is being regulated cannot be directly inferred from the gene expression data, but it appears to be complex. The best studied mechanism of DAF-16 regulation is repression through a phosphorylation cascade initiated by the binding of insulin-like ligands to the DAF-2 receptor [26,27]. However, we observed that the *ins-7* gene, which encodes a known agonist of DAF-2, is up-regulated in response to dichlorvos exposure (Figure 7B) which should result in the suppression of DAF-16 activity rather than the inferred activation. This observation suggests that there is a signal activating DAF-16-mediated transcription that is independent of DAF-2 and possibly countering the normal repression elicited by INS-7/DAF-2 binding.

A likely mechanism for activation of DAF-16 is evident from the elevated ADP/ATP ratios that have been documented as a result of organophosphate exposure [28]. It is possible that one of the AMP-kinases—which are sensitive to ADP/ATP balance—is transducing this energy metabolism signal to DAF-16, a known AMPK target [29]. Consistent with this possibility, we found that genes known to be up-regulated in *crh-1* mutants, an ortholog of the cAMP-inducible CREB transcription factor and downstream target of AMPK signaling [30], were over-represented in the microarray cluster analysis (Additional file 2) and primarily up-regulated in response to dichlorvos. AAK-2 is a strong candidate for the AMPK that could be activating DAF-16, as genes downstream of *aak-2* are over-represented in the dichlorvos data set (Additional file 2); furthermore, the gene encoding superoxide dismutase, SOD-3, a known DAF-16 target, is down-regulated in AAK-2 mutants [29].

The AMP dependent kinase, KIN-29 is a less likely candidate for activating DAF-16. In response to dichlorvos exposure, the message levels for KIN-29 are up-regulated. Further, it has been shown to inactivate TORC2 [31], an essential transducer in the DAF-2 signaling pathway. Although, KIN-29 may play a role in metabolic signaling since it is involved in food sensing, it appears to be chiefly involved in regulating chemoreceptor signaling in sensory neurons [32], with limited expressions elsewhere in the nervous system and in body wall muscles and hypodermal cells [33]. These factors argue against KIN-29 playing an important role in DAF-16 regulation.

In our analysis, we have inferred that DAF-16 is activated based on the behavior of the genes proposed as downstream targets of DAF-16 by Murphy *et al.*[25]. However, approximately 27% of the putative DAF-16 target genes in our study are not regulated in the direction that would be predicted from Murphy and coworkers’ experiments [25]. Functional analysis did not reveal any specific functions for the discrepant set of genes that were distinct from the functions observed for the complete set of differentially expressed genes. We did, however, observe that this set was enriched with genes containing PQM-1 binding sites (FDR = 0.01), suggesting an alternative transcriptional regulator event that might be responsive to reactive oxygen stress (see below).

In our view, the differences in results in the studies most likely arise from differences in the experimental designs, particularly because it is not possible to distinguish between direct and indirect effects, or among effects due to differential activity of the DAF-16 isoforms [26,34]. Murphy and coworkers used adult worms fed on a bacterial lawn in a purely genetic experiment, while we studied larval worms in axenic medium exposed to a chemical toxicant. It is known that regulatory processes involving DAF-16 are complex, and the differences in life stage of the worm, culture conditions, and experimental perturbation could easily account for the differences we see. We conclude that the effects we have described are largely DAF-16 mediated in response to dichlorvos exposure.

Based on our observations, we believe that the DAF-16 mediated gene response is a result of reduced energy reserves within the cells and is not a DAF-2 mediated event. Two known mechanisms of OP toxicity can contribute to this effect: 1) elevated consumption of energy because of increased muscular activity due to continual stimulation when acetylcholine is not degraded and 2) cholinergic receptor-independent mitochondrial disruption [28]. Furthermore, in other work, worms exposed to similar dichlorvos concentrations show reduced bacterial feeding [35]. These effects, which are likely additive, appear to be driving forces in DAF-16 mediated gene expression changes affecting metabolic processes.

#### Innate immunity related responses

We found a number of similarities between responses elicited by dichlorvos and infection in *C. elegans.* Dichlorvos exposure appears to activate some innate immunity signaling cascades (see below), and microarray cluster enrichment analysis (Additional file 2) revealed that many genes affected by dichlorvos exposure are in data sets derived from worms infected with a diverse set of pathogens and show similar regulation. However, dichlorvos exposure does not fully mimic the responses to infection.

*Microbacterium nematophilum*, *Enterococcus faecalis*, *Photorhabdus luminescens*, *Yersinia pestis*, and *Pseudomonas aeruginosa* infections activate many of the same genes as dichlorvos, and show similar regulation. Many of these genes are known to be involved in innate immunity and were up-regulated in response to the exposure, including genes encoding lysozymes (*lys-3* and *lys-7*), invertebrate lysozymes (*ilys-2* and *ilys-3*), antibacterial factors (*abf-2* and *abf-5*), caenacin antimicrobial peptides (*cnc-2*, *cnc-3*, *cnc-4*, and *cnc-6*) and 15 C-type lectins. The response appears to overlap but to be distinct from innate immunity because other genes considered to part of the innate immune response are down-regulated, including *lys-1*, *lys-2*, *lys-5*, *lys-6*, *ilys-5*, and 24 C-type lectins genes.

Dichlorvos exposure also impacts three of the seven signaling cascades known to be involved in immune defense in *C. elegans*[36]: the DAF-2 insulin-like receptor pathway (see above), the SMA/TGB-β pathway, and the p38/PMK-1 MAPK pathway. The components of the SMA/TGB-β pathway—the TGF-β receptor (*sma-6*), *sma −4* (a dwarfin signaling molecule) and *sma-10* (an LRIG family transmembrane regulator of SMA-6)—are all up-regulated.

The effects of dichlorvos on the p38/PMK-1 MAPK and related pathways are complicated. We observed up-regulation of the Toll-domain containing receptor (*tir-1*) upstream of p38/PMK-1 along with *sek-1*, the MAP kinase kinase regulator of PMK-1, and *pmk-1*. We also found enrichment for genes with a binding site for the GATA-family transcription factor, ELT-3, which has been shown to affect PMK-1 mediated expression of antimicrobial peptides [37]. The expression of *pmk-1* itself is regulated by NPR-1, a homolog of the neuropeptide Y receptor, and we find that 17 of the 27 NPR-1 regulated genes [38] present in our array data (enrichment *p = 10*^*-7*^) are differentially expressed.

However, the direction of gene change induced by dichlorvos for PMK-1 responsive genes and for NPR-1 regulated genes is primarily in the opposite direction from those predicted for PMK-1 mediated innate immunity [38,39]. A potential reason for this discrepancy is the increased expression of the gene encoding the phosphatase VHP-1, which is a known antagonist of PMK-1 [40]. VHP-1 is a dual-specificity phosphatase that interacts with multiple MAP kinases including KGB-1, PMK-3, and PMK-1 [40-42] and plays a role in modulating the respective signaling pathways during response to stress. It has been shown to be regulated by RNT-1 during oxidative stress [41]. It is likely that the increased expression of *vhp-1* and the concurrent repression of the PMK-1 pathway are the results of dichlorvos-induced stress.

The role of the DAF-2 insulin-like pathway in the immune response triggered by dichlorvos is somewhat complex. As mentioned above, the DAF-2 pathway is presumably being activated by INS-7, however, a secondary signal, likely via an AMPK, is preventing DAF-2 mediated suppression of DAF-16 activity. What makes this more intriguing is that the induction of *ins-7* is required for *P. aeruginosa* virulence [43], presumably due to suppression of the immune response. Three other genes encoding insulin-like peptides (*ins-4*, *ins-23*, and *ins-37*) are also up-regulated and one (*ins-33*) is down-regulated, although all are affected to a much lesser extent than *ins-7*. With the variety of signaling molecules and control mechanisms acting on the DAF-2 pathway, it is difficult to determine whether the observed effects are part of an immune response or represent another role of the DAF-2 signaling pathway. It is clear that the pattern of expression for some of these genes overlaps with the immune response.

There has been some discussion in the community as to whether the *C. elegans’* immune response is specifically triggered by pathogenic signaling molecules or if it is a generalized stress response [44], since no homologs for several key components of the mammalian Toll-like receptor pathway have been identified in the worm. Under the axenic culture conditions utilized in this work, the differentially expressed genes that are involved in the immune response are not being induced by a pathogen. However, it is not clear whether the dichlorvos stimulation of the innate immunity response acts through a “normal” stress sensing process or whether it is affected by an aberrant “toxic” mechanism. There are four primary mechanisms by which dichlorvos could be triggering the innate immune response: generalized stress, altered feeding pathways, autogenic stimulation, or neuronal signaling.

The simplest explanation for stimulation of the immune response is through stress pathways induced by the dichlorvos exposure, but others must also be considered. Since bacteria are the primary food source for *C. elegans* under natural conditions, there are overlaps between the immune response to bacteria and adaptation to different bacterial food sources [45]. Therefore, the adaptive response triggered by the low energy state within the worms might induce genes necessary for feeding on different bacteria, and these genes may contribute to the observed immune response. Another possibility has recently been described in mammals. Molecules present in organelles derived from ancient prokaryote symbiotes have characteristics that cause them to trigger immune receptors in the same manner as infectious bacteria [46]. In light of the potential damage to muscle and nerves (see below), the release of these molecules is likely to be occurring and might be the cause of the immune response induced by dichlorvos exposure; though it is still unclear whether *C. elegans* responds to these molecules in this way [44]. Finally, the primary mechanism of dichlorvos toxicity, the inhibition of AChE, may disrupt the neuronal signaling that helps to control the innate immune response [38,47]. It is reasonable to assume that with the variety of genes and processes that comprise the immune response that the stimulation of this response seen in exposed worms includes a combination, if not all, of these processes.

#### Neural and musculature repair responses

The gene expression changes seen at the later time points (14–26 h) highlight the known myopathic and neurodegenerative effects of organophosphate poisoning [48,49] and are consistent with increased muscular and neuronal growth and repair. There was an increase in expression levels for a large number of muscle-specific genes that are required for myogenesis. Genes encoding proteins localized to the M-bands (Additional file 3) are over represented in a gene ontology analysis; 12 of the 18 genes present in the data set that encode M band proteins are up-regulated. Other muscle genes up-regulated include those encoding titin (*ttn-1*), two paralogs of titin (*ketn-1* and *unc-22*), homologs of troponin T and C (*mup-2* and *tnc-2*), and the homolog of tropomyosin (*lev-11*).

The evidence for neuronal growth and repair is most apparent through effects that are related to axon guidance and regeneration. Two genes in the DLK-1 MAPK pathway, *dlk-1,* a MAPKKK, and *pmk-3*, a p38 MAPK, were up-regulated. The DLK-1 pathway is essential for axon regeneration [50]. There was also increased expression of multiple genes required for axonal guidance (*unc-14*, *unc-129*, *eva-1*, *klc-2*, and *pak-1*). In addition, two of the pathways that are involved in innate immunity play a dual role in the worm and are also involved in neuronal cell fate determination. The DBL-1/SMA TGF-β pathway has been implicated in controlling neuronal cell fate in the male sensory array [51], and the proteins encoded by two of the genes up-regulated in the PMK-1 MAPK pathway, UNC-43 and SEK-1, function to determine asymmetric cell fate decision during sensory neuron development [52,53]. It is not possible to conclude from the data in-hand whether or not the changes in these pathways are the result of innate immunity or cell fate determination, or if they may be involved in both.

Taken together, these gene expression changes suggest that at the later time points in the exposure, the worms have significant cellular damage and have initiated multiple repair processes. The mechanism by which dichlorvos provokes myopathic and neurodegenerative effects has not been defined, but some work suggests that it is related to mitochondrial dysfunction [54]. The changes in metabolic processes and potential mitochondrial dysfunction (see below) observed in response to dichlorvos exposure make this a plausible mechanism for the damage induced in the worms.

#### Early responses

**Figure 8:**
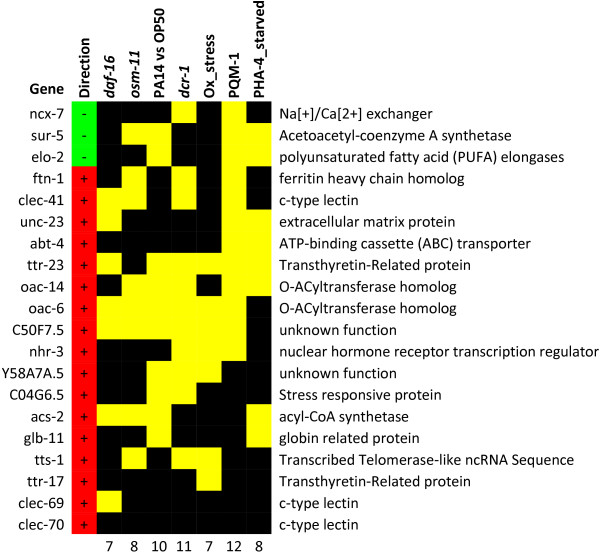
**Enrichment analyses of the 20 genes found to be differentially expressed after 2 h of exposure to dichlorvos were performed (Additional files****2****,****3****,****4****).** Columns include experiments where enrichment was observed for at least one condition. Lists from microarray clusters for up and down-regulated genes were merged for *daf-16*[25], *osm-11*[55], and *dcr-*1 [56], Ox_stress [57] and also for time after infection for PA14 vs OP50 [39]. PQM-1 and PHA-4_starved are list of genes with binding sites for the respective transcription factors [24]. Cells marked with yellow indicate dichlorvos-responsive genes that are present in the gene set indicated at the head of the table. The numbers at the foot indicate the number of genes shared between the 2 h dichlorvos exposure gene set and the indicated experimental data. The direction of change in gene expression is shown in the left-most column, minus sign/green—down-regulated, plus sign/red—up-regulated.

In order to focus on the primary effects of dichlorvos intoxication, we closely examined the 20 genes that were differentially expressed within the first 2 h of the exposure (Figure 8). The most notable biological processes affected at this early time point are immune response and energy metabolism. In the microarray cluster enrichment analysis, this was highlighted by over representation of genes regulated by DAF-16 or differentially expressed during infection by *P. aeruginosa* (Additional file 2). The down-regulation of the genes encoding a fatty acid elongase (*elo-2*) and an acetoacetyl-coA synthetase (*sur-5*) suggest a reduction in fatty acid elongation, while the up-regulation of the gene encoding an acyl-CoA synthetase (*acs-2*) implies an increase in β-oxidation.

Over half of the genes differentially expressed at this time have a binding site for PQM-1 (Additional file 4), a transcription factor strongly up-regulated by paraquat exposure, and seven of the genes were identified as being up-regulated by oxidative stress (Additional file 2). The primary mechanism of paraquat toxicity is believed to be reactive oxygen species (ROS) generation mediated by mitochondria [58]. Taken together, these observations suggest that the early effects of dichlorvos exposure include a reduction of available energy and increased ROS. While increases in β-oxidation contribute to ROS generation, the gene response suggests a higher level of ROS than would be anticipated by β-oxidation alone. A lower energy state and higher levels of ROS are consistent with the idea that dichlorvos causes mitochondrial dysfunction.

The reason for overlap with genes differentially regulated during *P. aeruginosa* infection is not as directly evident. There are no bacteria present, and it is unlikely to be autogenic due to damaged cells. The two most plausible reasons are 1) a stress-mediated immune response, or 2) overlap with non-immune mediated DAF-2 insulin signaling. The latter is of particular relevance considering one of the pathogenic mechanisms of *P. aeruginosa* is the up-regulation of *ins-7*[43] and while *ins-7* was not differentially expressed at 2 h based on our strict criteria, it is in fact modestly up-regulated (1.2 fold) and strongly up-regulated at later time points (Figure 7B). Therefore, the genes regulated by *P. aeruginosa* infection are likely to include genes regulated by the DAF-2 insulin signaling pathway both in an immune-specific and non-specific manner.

The majority of the genes differentially expressed as an early response to dichlorvos is directly related to energy metabolism or is regulated by the DAF-2 insulin-signaling pathway. These effects highlight a change in metabolism within the worms that is indicative of reduction in available energy and the utilization of energy reserves. The inclusion of genes regulated by oxidative stress and PQM-1 hints at mitochondrial dysfunction as the mechanism for the reduction in energy levels.

### Recovery

An important goal of this study was to identify genes and biological processes that might be involved in recovery from dichlorvos intoxication. To identify genes that participate in the recovery process and not ongoing toxicity, data were examined from unexposed and high concentration samples collected after the 8 h washout (14–26 h harvests). We used the same criteria for selecting differentially expressed genes in the washout conditions [ANCOVA (PA and exposure) *p* < 10^-4^ and > 1.8-fold change] that we used for the continuous exposure analysis. A total of 78 probe sets meet these criteria for differential expression in at least one of the harvest times.

**Figure 9:**
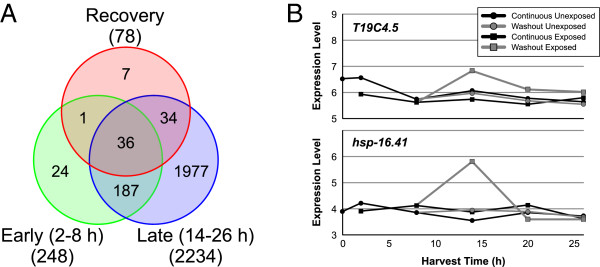
**Worm recovery after removal of toxicant. A**) Venn diagram of differentially expressed probe sets in washout samples after an 8 h exposure compared to probe sets differentially expressed early (2–8 h) or late (14–26 h) in the continuously exposed worms. **B**) Plot of expression level versus time for two genes uniquely differentially expressed during recovery phase

The effect of the exposure diminishes rapidly with time as there are 74 probe sets differentially expressed in the 14 h washout samples and only 7 at the 20 h time point. There were no significant differences at the 26 h time point. As expected, many of these probes are also differentially expressed in the continuous exposure regime (Figure 9A). An interesting observation is that 34 probe sets that were not identified as being differentially expressed at the 8 h time point are differentially expressed at later times by the worms in both the continuous exposure and washout protocols, although the magnitude of differential expression is lower for nearly every probe set in the washout samples than the continuously exposed samples. While trending suggests that some of these genes are affected at 8 h (though not statistically significant according to our criteria), there is a set of genes whose expression is clearly affected only after the washout has occurred. This observation suggests that there are processes affected by dichlorvos which produce delayed effects even after removal of the toxicant. We were unable to find any statistically enriched processes related to these genes, but many appear to be involved in immune response and energy homeostasis.

The probe sets differentially expressed in the washout samples, but not the continuous exposure regime, are the most attractive targets for identifying recovery-specific effects. There are seven probe sets that were identified only in the washout samples. However, because the selection criteria were designed to stringently minimize false positives, it is possible that some of them actually could be differentially expressed during the continuous exposure regime. We used less stringent criteria (p <0.01 and 1.5-fold change) to allow us to discern probe sets that were weakly differentially expressed in the continuous exposure regime and found that only two of the seven probe sets were uniquely differentially expressed after the washout procedure (Figure 9B). Unfortunately, the proteins encoded by the genes targeted by these probe sets, a widely expressed heat shock protein (HSP*-*16.41) and an iron-binding protein of unknown function (T19C4.5), offer little insight into recovery specific mechanisms.

In addition to looking at the genes that were uniquely identified in the recovery period, we examined the entire group of probe sets that were differentially expressed during the recovery phase to determine biological mechanisms potentially involved in recovery. This list was subdivided to include only the 41 probe sets that were differentially expressed using strict criteria after the dichlorvos was removed (i.e. they were not differentially expressed at the 8 h harvest). In both the complete list and the “after exposure” list, the biological processes that are represented appear to exactly mimic those that are seen during the exposure, albeit to a lesser extent (See Additional Files 2, 3, 4). No recovery-specific mechanisms were identified.

After removal of dichlorvos from the medium, the gene expression changes seen in the worms appear to return to baseline levels rather rapidly, and those observed at the first harvest after the washout simply appear to be residual effects of ongoing biological processes resulting from the exposure. By the 26 h harvest time, the study lacks sufficient power to identify any differentially expressed genes. With the exception of expression changes that appear to be the result of delayed development, the treated worms appear to have fully recovered from the exposure.

### Mechanism of toxicity

Many if not all of the biological effects identified in this work that were caused by dichlorvos exposure can be plausibly tied to inhibition of acetylcholinesterase. Developmental timing in *C. elegans* requires active nicotinic acetylcholine receptors [59]. Persistent cholinergic signaling causes a depletion of muscular ATP that can mediate an increase in certain metabolic processes [60]. Acetylcholine signaling during starvation activates a MAPK (MPK-1) in pharyngeal muscles [61]. Innate immunity in *C. elegans* is known to be at least partially regulated by neuronal cells and signaling [38], and this signaling leads to the activation of PMK-1 [47]. From these examples, it is clear that neuronal signaling and acetylcholine levels can affect metabolic processes, innate immunity, and developmental time, which are the primary effects observed in response to dichlorvos exposure.

Though AChE inhibition is likely the dominant cause of toxicity in *C. elegans*, there are two other non-cholinergic mechanisms that have been implicated in dichlorvos toxicity that are consistent with the observations in this work; they are mitochondrial dysfunction [28] and perturbation of calcium homeostasis [62].

The metabolic changes highlighted by alterations in expression levels of DAF-16 target genes underscore the lower energy reserves present in exposed worms. Some of this deficiency may be caused by increased muscle stimulation or reduced feeding, but the high levels of induction suggest a more extreme reason for this deficiency, such as a reduction in energy production triggered by the effects of dichlorvos on mitochondrial function. The down-regulation of the genes encoding multiple subunits of the mitochondrial electron transport chain including ones from complex I (*gas-1*, *C18E9.4*, and *D2030.4*), complex II (*sdha-1*), and complex V (*asg-2*) suggests a reduction in mitochondrial energy production. Further work will be required to delineate what roles mitochondrial dysfunction is playing in dichlorvos toxicity.

The role of calcium homeostasis in dichlorvos toxicity is another area where the data do not provide a clear answer but are very suggestive. There are numerous calcium dependent processes that are perturbed by the exposure. For example, the gene encoding UNC-43, a calmodulin-dependent protein kinase that can activate the p38/PMK-1 MAPK pathway, is up-regulated and could be the cause of the activation of that pathway seen in this work. There is also evidence for activation of a calcineurin pathway. The gene encoding CNB-1, an ortholog of calcineurin B and *rcn-1,* a gene whose transcription is dependent on a calcineurin A ortholog (TAX-6) and free calcium, are both up-regulated. Furthermore, genes known to be regulated by TAX-6 are over represented in the data set (Additional file 2). The exact role of calcium homeostasis in dichlorvos toxicity is unknown, and there is no defined mechanism by which dichlorvos effects calcium homeostasis. This is another promising area where more work must be performed to fully understand the effects of dichlorvos toxicity.

## Conclusion

Upon exposure to dichlorvos, worms show a time-dependent response with progressive delays in development, alterations in metabolism, increased cellular repair, and alterations in innate immunity functions. Early in the course of the exposure, the effects appear to be primarily related to energy metabolism. As the exposure progresses, the changes in the metabolic pathways become more pronounced, and there is increased perturbation of immune responses. At the latest times, repair processes have been activated that are presumably a response to damaged nerve and muscles cells. Upon removal of the toxicant, gene expression quickly returns to baseline, with the only lingering effect being a synchronized delay in developmental gene expression. This delay in development is the single largest effect on the worms and is evident as early as 8 h after treatment.

There are several potential mechanisms by which dichlorvos is mediating toxicity in *C. elegans*. Inhibition of AChE is likely a key player as many of the perturbed processes involve neuronal signaling, and the observed behavior changes are consistent with increased accumulation of acetylcholine. The observed changes in the expression of genes involved in energy metabolism suggest that dichlorvos is disrupting mitochondrial function, which has been shown to be a mechanism for neuronal degeneration [54]. Calcium homeostasis is potentially another process perturbed by dichlorvos exposure, but it is unclear whether dichlorvos directly or indirectly perturbs calcium homeostasis.

**Figure 10:**
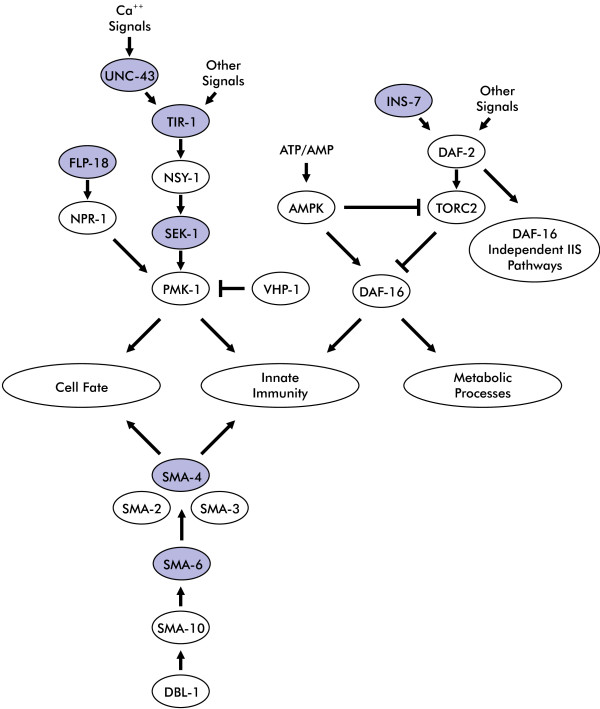
**Key pathways that are affected by dichlorvos exposure.** Genes highlighted in blue are up-regulated in response to dichlorvos

Through this work, we have identified key genes and pathways responding to dichlorvos toxicity (Figure 10). The primary mechanisms appear to be inhibition of AChE and depletion of energy reserves possibly through mitochondrial dysfunction. We have also shown the resilience of the worms by their recovery after removal of the toxicant. This work provides insight into the detailed process of dichlorvos intoxication and sets the groundwork for future efforts.

## Methods

### Culture

The basic culture conditions have been described previously [17,63]. Briefly, all experiments were performed using N2 wild type *C. elegans* [DR subclone of CB original (Tcl pattern I)], obtained from the Caenorhabditis Genetics Center, (Saint Paul, MN) and cultured in CeHR medium at 22.5 °C in T-75 tissue culture flasks with shaking at 70 rpm. CeHR medium is an axenic, sterile, defined liquid medium containing 20% ultra pasteurized organic milk and described in detail previously [63]. The development of the cultures was synchronized using the alkaline hypochlorite method of Hope [64] with minor modifications described in Szilagyi *et al.*[63]. Egg counts were determined using a hemocytometer.

### Range-find

In previous work [17], 15 μM dichlorvos in CeHR medium produced readily assayable changes in global gene expression in worms exposed for 8 h during the L4 larval stage. To maintain consistency, 15 μM was set as the high concentration for the work at hand. We determined the lower concentration for this experiment by observing the response of the worms to four concentration of dichlorvos from 15 μM to 0.12 μM using five-fold dilutions. Worm development and mobility were monitored by microscopy over a 24 h period of continuous exposure beginning at the mid-vulval L4 stage (46 h post synchronization). In addition, cultures of worms were exposed to dichlorvos for 4 h, washed three times with medium lacking milk to remove the dichlorvos, and returned to the incubator in medium to ascertain whether the worms could resume development and regain motility after exposure. Similarly treated unexposed cultures were used as controls. Observations of the continuously exposed cultures were made at 2, 3, 5, 6, 7 and 24 h after dosing and of the washed out cultures at 1, 2, 3 and 20 h after removal of the dichlorvos. The observations are summarized in. The lowest concentration showing effects on movement (0.6 μM) was then used as the low concentration for the definitive exposures.

### Exposures

The time course for the definitive experiment is diagrammed in Figure 1. Cultures of 250,000 synchronized L1 worms were grown in 30 mL CeHR medium as described above. After ~41 h, when 50% of the worms have passed the L3/L4 molt, we commenced the exposure. The molt coincides with early turning of the gonadal arms and the appearance of a vulval slit approximately one cell width wide. The developmental stage of several representative flasks of worms was confirmed microscopically before commencing the experiment.

In order to eliminate effects caused by changes in volume due to repeated sampling from the flasks over the course of the experiment, we set up each condition in its own T-75 culture flask. Four flasks of worms were harvested prior to beginning the exposure as the 0 h controls. Equal volumes of water or dichlorvos stock (diluted for 0.6 μM or 15 μM final concentration) as appropriate were added to the remaining flasks, which were then returned to the incubator. The flasks were treated according to one of three protocols. Untreated controls (or shams) and low and high concentrations flasks were prepared for each harvest time for each of the three protocols. In the first protocol, the set of flasks was incubated without interruption for the duration of the experiment with flasks being harvested at 2, 8, 14, 20, and 26 h. The other two sets were incubated for 2 or 8 h, after which the worms were centrifuged out of the exposure medium, washed three times with Washout Buffer, a modified CeHR medium (see below), resuspended in fresh CeHR medium without dichlorvos, and returned to the incubator. Flasks were harvested at 6 h intervals following the washout through 26 h.

To minimize effects on normal worm development and gene expression and to prevent the accumulation of insoluble components, including milk solids, which would result from repeated washes and centrifugation with standard CeHR medium, the 30–45 min washout procedure was performed using a modified CeHR medium (Washout Buffer). Fat-free, ultra-pasteurized organic milk was repeatedly centrifuged, and the supernatant was sterile-filtered to produce clarified milk. Clarified milk (20% v/v) was added to the following components at the same concentrations as standard CeHR medium [63] to make Washout Buffer: lactalbumin, essential amino acids, non-essential amino acids, KH_2_PO_4_, HEPES, and glucose. The osmolarity and pH of Washout Buffer are similar to those of CeHR medium.

### Chemistry

As described previously [17], stock solutions of dichlorvos (Chem Service, West Chester, PA) in water were prepared weekly, filter-sterilized, and the concentrations were verified. The dichlorvos concentrations were stable within 10% over 24 h. Dichlorvos concentrations were determined using a minor variation of EPA method 8141A and a Hewlett-Packard model 6890 gas chromatograph equipped with an electron capture detector and a Hewlett-Packard model 7673 auto sampler (Santa Clara, CA).

### RNA extraction, processing, and labeling

Worm harvesting, RNA extraction, processing and labeling were performed as previously described [17]. Briefly, worms were harvested by centrifugation, washed with 100 mM NaCl, frozen in liquid N_2_, and stored at −80 °C until use. The frozen worms were pulverized under liquid N_2_ in a Spex 6750 Freezer Mill (Metuchen, NJ). RNA was extracted from the pulverized worms with Trizol solution (Invitrogen / Life Technologies, Grand Island, NY) followed by an additional purification step using the RNeasy Midi Kit (Qiagen, Valencia, CA). Poly(A)+ RNA was isolated using OligoTex (Qiagen). cDNA was synthesized using the SuperScript Choice kit (Invitrogen) and a T_24_T7 primer. Biotin labeled cRNA was synthesized using the Enzo BioArray High Yield kit (Farmingdale, NY).

As recommended by Affymetrix, cRNA samples were hybridized to Affymetrix *C. elegans* whole genome GeneChips, processed, and scanned by the Laboratory of Dr. Maryanne Vahey, Division of Retrovirology, Walter Reed Army Institute of Research.

### Microarray analysis

Standard Affymetrix-recommended quality control parameters were used. In addition, replicate samples were compared after RMA normalization [65] using multivariate correlation, and replicate vs. replicate dot plots. Replicates were accepted if they passed Affymetrix recommended standards, had an R^2^ ≥ 0.93, and displayed no gross deviations from linearity in dot plots.

The Bioconductor implementation of the Affymetrix MAS 5.0 algorithm in the R statistical package was used to generate Present/Marginal/Absent calls for all probe sets on each microarray. Probe sets were considered “Present” and retained for further analysis if they were called Present in all replicates of at least one condition. This procedure reduced the total number of probe sets under consideration to 14,398 from the approximately 22,500 on the microarray. Statistical analysis and data processing (including RMA) were performed using Partek Genomics Suite (Partek, Inc., St. Louis, MO).

Microarray data have been deposited in the Gene Expression Omnibus [66], Accession Number GSE41366.

#### Developmental delays

In the course of this and earlier studies, we observed that chemical exposures (including dichlorvos exposure) generally resulted in the slowing of the development of the worms (see Results). To control for changes in gene expression due to developmental delay, worms were “staged” based on expression patterns of developmentally regulated genes identified in unexposed worms using linear regression models. In the data from the unexposed, no washout samples, we found 6,132 probes sets that were significantly different by harvest time in a one-way ANOVA [False Discovery Rate (FDR) < 0.001] and that changed by at least 1.8 fold in abundance from time zero to at least one other harvest time.

A subset of these probe sets was selected for each harvest time to use in the linear regressions. The subset included only probe sets that showed at least 2-fold change in abundance over the preceding two harvest intervals and had a linear response (r^2^ > 0.95). We then excluded probe sets that showed dichlorvos-dependent changes in gene expression; in this way, we were able to define a group of developmentally-regulated, dichlorvos-independent probe sets.

Using data from the continuous, unexposed condition, we produced a set of equations for predicting apparent developmental age from gene expression by performing a linear regression between harvest time and expression level for each member of this developmentally-regulated, dichlorvos-independent collection of probe sets. The average of all the apparent ages for all of the probe sets is the Predicted Age (PA) of each sample; the start of the exposure was set as age zero.

#### Toxicant specific gene changes

We identified genes that showed dichlorvos-dependent, developmentally-independent changes in expression by comparing the high concentration and control samples from the continuous exposure regimen. We performed ANCOVA tests using the PA and exposure as factors and included contrasts to calculate fold-change. Separate analyses were performed for each harvest time using the data from the exposed samples from each harvest time along with the controls from that harvest and the preceding harvest. A Benjamini and Hochberg FDR [67] of < 0.001 across the five harvest times corresponded to a *p* < 10^−4^.

Differentially expressed probe sets were selected using a *p* < 10^−4^ and a 1.8 fold change filter. To eliminate false positives that might have been introduced by large changes in expression and a non-linear response—that is assumed by the statistical test—across a harvest interval, we retained only probe sets with expression levels that were at least 1.2 fold outside of the range of expression levels seen in the control samples from the harvest times that were included in the ANOVA.

### Functional analysis

In order to assist in functional interpretation of the microarray data, enrichment analyses were performed with publically available data sets to identify gene sets that were statistically significantly over-represented in our differentially expressed gene lists. Probe sets were mapped to genes using WormBase release WS224, and only those probe sets mapping to a single gene and “Present” above the signal to noise threshold (see above) were considered in the enrichment analysis. This included 13,111 probe sets targeting 10,677 genes. Lists of genes were downloaded from WormBase release WS227 using an AQL (ACEDB Query Language) Query for all Gene Ontology Terms and Microarray Expression Clusters and from the publisher’s website for transcription factor binding sites [24]. Using a custom written Perl script, each publically available gene list was compared to the differentially expressed gene lists to identify overlapping genes. The probability for a given number of overlaps between lists occurring by chance was computed using a cumulative hypergeometric distribution; a Benjamini-Hochberg step-up FDR [67] was used to control for multiple tests. For all data sets except those that were derived from Affymetrix microarrays, the population size was considered to be the 10,677 genes, which had “Present” probe sets that targeted a single, known gene. For data from Microarray Expression Clusters resulting from experiments using Affymetrix *C. elegans* microarrays, the 13,111 probe sets were used as the population for calculating enrichment. Gene sets with an FDR < 0.01 in any one condition were further examined in all conditions. The enrichment results can be found in Additional files 2, 3, 4. In several cases, DAVID Bioinformatic Resources [23,68,69] were also used to assess whether genes with particular gene ontology terms occurred more frequently than expected by chance.

## Abbreviations

AChE: Acetylcholinesterase; ANCOVA: Analysis of covariance; ANOVA: Analysis of variance; AQL: ACEDB Query Language; FDR: False discovery rate; LOEL: Lowest observed effect level; NTE: Neuropathy target esterase; OP: Organophosphate; OPIDP: Organophosphate induced delayed polyneuropathy; ROS: Reactive oxygen species

## Competing interests

The authors have no competing interests to declare.

## Authors’ contributions

CEB performed experiments. EAG designed and performed experiments. JAL conceived, designed, and performed experiments, analyzed data, and wrote the paper. DAJ conceived and designed experiments, analyzed data, and wrote the paper. All authors read and approved the final manuscript.

## Supplementary Material

Additional file 1 Developmentally Regulated Genes Whose Timing of Expression is Unaffected by Dichlorvos-induced Developmental Delays. Click here for file

Additional file 2 Results of microarray clusters enrichment analysis. Click here for file

Additional file 3 Results of gene ontology enrichment analysis. Click here for file

Additional file 4 Results of binding site enrichment analysis. Click here for file
