# Supplementary material for: Alterations in gene expression in Caenorhabditis elegans associated with organophosphate pesticide intoxication and recovery
Source: BMC Genomics. 2013 Apr 30;14:291. doi: 10.1186/1471-2164-14-291 (PMC3760450; doi:10.1186/1471-2164-14-291)
Supplement: Additional file 4 — Results of binding site enrichment analysis. [file 1471-2164-14-291-S4.pdf]

Developmentally Regulated Genes Whose Timing of Expression is Unaffected by Dichlorvos-induced Developmental Delays

Oligo_set	WBGeneID	Gene	Name
192458_at	WBGene00000002	aat-1	Amino Acid Transporter
174095_at	WBGene00015484	C05D11.7	
174170_at	WBGene00015484	C05D11.7	
174896_at	WBGene00007593	C14H10.3	
184919_s_at	WBGene00017992	F32E10.5	
182110_at	WBGene00018829	F54E7.6	
185731_at	WBGene00019112	F59E11.6	
184953_at	WBGene00013755	fbxa-115	F-box A protein
181521_at	WBGene00017904	lim-8	LIM domain family
174319_at	WBGene00016955	perm-5	PERMeable eggshell
180355_at	WBGene00016955	perm-5	PERMeable eggshell
181621_at	WBGene00012002	T24H10.4	
192032_s_at	WBGene00015168	tag-320	
174566_s_at	WBGene00012221	W03C9.5	
180920_s_at	WBGene00014194	ZK1037.6	

°
